# Artificial Intelligence Chatbots (ChatGPT and Google Gemini) Versus Traditional Patient Information Leaflets for Local Anesthesia in Eye Surgery: Correspondence

**DOI:** 10.22599/bioj.423

**Published:** 2024-11-06

**Authors:** Hinpetch Daungsupawong, Viroj Wiwanitkit

**Affiliations:** 1 Lao people’s democratic republic, LA; 2 Department of Research Analytics, Saveetha Dental College and Hospitals, Saveetha Institute of Medical and Technical Sciences Saveetha University, Chennai, India

**Keywords:** artificial intelligence, Chatbot, anesthesia

Dear Editor,

We would like to discuss on ‘Comparative Analysis of Accuracy, Readability, Sentiment, and Actionability: Artificial Intelligence Chatbots (ChatGPT and Google Gemini) versus Traditional Patient Information Leaflets for Local Anesthesia in Eye Surgery (Gondode et al., 2024).’ ‘Frail Older Adults’ Needs and Preferences for Mobile Health Exercise Interventions Guided by Nudge Theory: A Qualitative Analysis’ [1]. This study conducted a comparative analysis of patient education documents generated by AI chatbots (specifically ChatGPT and Google Gemini) with traditional patient information documents (PILs), focusing on the use of local anesthesia in eye surgery. The evaluation was conducted by expert reviewers who assessed the documents based on several criteria: accuracy, completeness, readability, sentiment, and understandability. The results indicated significant differences between the sources. While the AI chatbot presented information in a more accessible language, traditional PILs scored higher on accuracy and completeness. In particular, ChatGPT outperformed Google Gemini on accuracy and completeness, although PIL maintained the highest overall score.

Despite the detailed approach taken in this investigation, there are some glaring flaws. First, relying on expert reviewers may add subjective bias because their ratings may reflect personal views of readability and tone. Furthermore, the number and diversity of the materials examined may restrict generalizability. A broader range of chatbots and PILs should be included for a more complete comparison. Furthermore, this study may have failed to reflect the dynamic nature of AI-generated content, which can evolve quickly and have an impact on the results’ lifespan. Content assessments based on a single point in time may fail to account for changes in practices or AI language model modifications.

The approach used in this investigation is similarly limited. The study used statistical analysis, such as ANOVA and Tukey HSD tests to compare the performance of various sources. While these assessments are useful, they may be insufficient to explain disparities in users’ experiences when reading educational information. Personal experiences with understanding and information recall, for example, can be influenced by factors such as prior knowledge and cognitive biases that are not taken into account in the assessment. Furthermore, focusing just on particular dimensions (accuracy, completeness, readability, tone, and comprehension) may result in overlooking other crucial characteristics, such as cultural sensitivity, visuals or multimedia integration, and information utility.

Future research could address these methodological limitations by soliciting feedback from a broader spectrum of users, such as through surveys or focus groups with patients and healthcare professionals. Furthermore, longitudinal studies that look at how AI-generated material adjusts over time in response to algorithm modifications or new data may provide useful insights. Examining a wider range of chatbot platforms and health themes may also aid in better understanding of AI in patient education. Furthermore, investigating the efficacy of these materials in real-world situations, such as clinical or post-operative settings, may help to identify best practices for their application.

This study is unique in that it compares AI-generated patient education materials to traditional PILs, a topic that has received little attention in healthcare research. The findings indicate fascinating benefits of AI chatbots in terms of accessibility and understandability, implying the possibility of hybrid approaches that combine the strengths of AI and traditional materials. For practitioners, these insights can help them build more successful patient education initiatives that use AI technologies while maintaining completeness and accuracy. Moving forward, creating hybrid tools that combine the simple language of AI outputs with the rigor of PILs could provide a novel way to improve patient understanding and participation with their care.
